# The Inflammatory Link of Rheumatoid Arthritis and Thrombosis: Pathogenic Molecular Circuits and Treatment Approaches

**DOI:** 10.3390/cimb47040291

**Published:** 2025-04-18

**Authors:** Theodora Adamantidi, Maria Stavroula Pisioti, Sofia Pitsouni, Chatzikamari Maria, Karamanis Georgios, Vasiliki Dania, Nikolaos Vordos, Xenophon Krokidis, Alexandros Tsoupras

**Affiliations:** 1 Hephaestus Laboratory, School of Chemistry, Faculty of Sciences, Democritus University of Thrace, Kavala University Campus, St Lucas, 65404 Kavala, Greece; theadam@chem.duth.gr (T.A.); mapisio@chem.duth.gr (M.S.P.); bopitso@chem.duth.gr (S.P.); 2 Hematology, Biochemistry and Internal Medicine Departments, General Hospital of Kavala, St Silas, 65500 Kavala, Greece; xatzifil@gmail.com (C.M.); biochemistry@kavalahospital.gr (K.G.); kroxen1959@yahoo.gr (X.K.); 3 First Department of Orthopedic Surgery, School of Medicine, National and Kapodistrian University of Athens, ATTIKON University General Hospital, 12462 Athens, Greece; viky_dania@yahoo.gr; 4 Biophysics Laboratory, School of Physics, Faculty of Sciences, Democritus University of Thrace, Kavala University Campus, St Lucas, 65404 Kavala, Greece; nvordos@physics.duth.gr

**Keywords:** rheumatoid arthritis, autoimmune diseases, inflammation, thrombosis, venous thromboembolic events, anticoagulants, antithrombotic therapy, anti-rheumatic drugs, DMARDs

## Abstract

Rheumatoid arthritis (RA) is a chronic autoimmune disease characterized by systemic inflammation that primarily affects the joints but can also involve extra-articular organs. Its multifactorial etiology remains incompletely understood, necessitating further investigation into its underlying mechanisms. The primary therapeutic goal in RA management is to achieve disease remission or maintain low RA activity to prevent long-term morbidity. RA therapies aim to mitigate joint damage, reduce disability, and prevent systemic complications such as cardiovascular diseases. In addition to pharmacological treatments, non-pharmacological interventions—including physiotherapy, occupational therapy, and lifestyle modifications such as smoking cessation, regular exercise, and adherence to a balanced diet—play a crucial role in managing the disease. Beyond joint inflammation, RA has been strongly associated with an increased risk of thrombosis, contributing significantly to both morbidity and mortality. The link between RA and thrombotic events arises from a complex interplay of inflammatory pathways, endothelial dysfunction, and coagulation abnormalities. This review provides an in-depth analysis of the mechanisms driving the association between thrombo-inflammatory manifestations and the incidence of RA, the impact of RA treatment on thrombosis prevalence, and potential therapeutic strategies for managing both conditions concurrently. By integrating recent advancements in rheumatoid arthritis (RA) pathophysiology and thrombo-inflammatory research, this paper provides a comprehensive resource on the inflammatory link between RA and thrombosis while discussing and comparing current and emerging treatment approaches. Further investigation into these mechanisms could facilitate the development of targeted therapies that reduce the risk of thrombosis in patients with RA.

## 1. Introduction

Rheumatoid arthritis (RA) is a well-known chronic autoimmune disorder that primarily affects the joints and is characterized by inflammatory arthritis. The exact etiology of RA remains poorly understood, with its pathogenesis involving a complex interplay of genetic, epigenetic, environmental, and immunological factors [1,2,3]. The origins of this disease can be traced back to the 19th century, and it has a “family tree” that evolved throughout the 20th century [4]. Although the term “rheumatoid arthritis” was introduced in the 1850s, its classification criteria were only developed approximately 50 years ago [1,2,4].

French doctors studied RA during the 19th century to better understand its pathophysiology and clinically test potential treatments, such as psychotherapy and balneotherapy (hot water baths) [5,6,7]. In 1948, glucocorticoid therapy was first introduced, reducing symptoms, while only a few years later, disease-modifying antirheumatic drugs (DMARDs) became available, slowing RA progression [8]. Until the 1990s, RA diagnosis had devastating consequences, often leading to progressive joint destruction, reduced life expectancy, and significant disability [9,10,11]. Additionally, primary asthenic gout, described as the first form of RA in 1800, was commonly observed [7].

In general, systemic inflammation linked to RA induces dysfunction beyond the joints, contributing to vasculitis, interstitial lung disease (ILD), and cardiovascular disease (CVD) risk [2,12,13,14]. The progression of this disease begins years before the appearance of symptoms in an individual. It is marked by the formation of specific autoantibodies, including rheumatoid factor (RF) and anti-citrullinated protein antibodies (ACPAs), by plasma cells [15,16]. Previous studies have highlighted that these autoantibodies primarily activate macrophages via Fc receptors. ACPA-positive patients are more likely to develop severe RA symptoms, an increased risk of CVD mortality, and potential atherosclerosis complications [2,15,16].

Various types of arthritis have been classified into two distinct categories: non-inflammatory arthritis (osteoarthritis) and inflammatory arthritis, which may be caused by crystal deposition, bacterial and viral infections, or autoimmune processes [17]. The disease affects women two to three times more often than men and can occur at any age, peaking in the sixth decade of life [18,19]. Early diagnosis and treatment of RA can prevent or significantly slow joint damage progression in up to 90% of patients, thereby reducing the risk of irreversible disability [18,20].

An increasing number of studies suggest that autoimmune diseases such as RA are risk factors for CVDs, as well as for venous thromboembolic events (VTE), including pulmonary embolism (PE) and deep vein thrombosis (DVT) [19,21,22]. RA patients have a higher predisposition to developing VTE due to common demographic factors, comorbidities, and lifestyle choices such as unhealthy dietary habits and smoking [19,23,24,25]. Furthermore, it is crucial to clarify that thrombosis—the formation of a blood clot within blood vessels—can occur in either venous or arterial vessels, thereby restricting natural blood flow and leading to clinical sequelae [22,26,27]. Thromboembolism can affect almost any blood vessel and is a complex, multifactorial condition that involves interactions between acquired or inherited thrombophilic predispositions and environmental factors [22,26,27].

The primary cell types implicated in thrombosis mechanisms include platelets, endothelial cells, and epithelial cells. Untreated VTE is commonly associated with high risk of recurrence and severe mortality rates [19,28,29,30,31]. As a result, anticoagulation therapy is considered the cornerstone of treatment for acute episodes and long-term prevention [17,32]. Antiplatelet approaches, on the other hand, are also explored as potential adjuvant therapies for preventing RA progression.

In addition to environmental risk factors for VTE, genetic and epigenetic factors—such as coagulation disorders and deficiencies in antithrombin, protein C, and protein S—also contribute to its pathogenesis [33]. RA patients share a notably higher risk of developing both arterial and venous thrombosis, with VTE risk estimated to be twice as high as in the general population [19,22,25,26]. The connection between RA and VTE is believed to be driven by systemic inflammation, with strong evidence indicating shared pathways between the innate immune system, coagulation mechanisms, and platelet activation. This may explain the interaction between inflammatory cytokines, platelet activation, and coagulation factors, as several molecular components are essential for immune and thrombotic responses [2,19,34,35,36].

**Figure 1:**
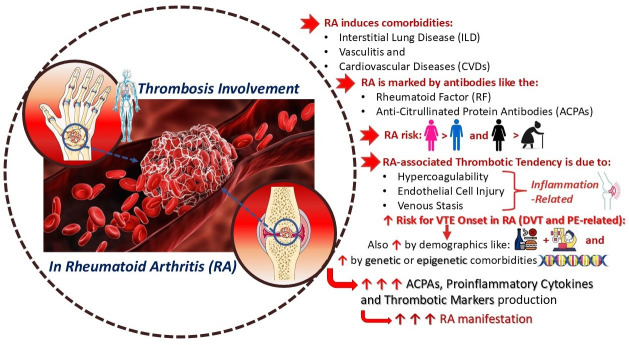
Thrombosis affiliation with rheumatoid arthritis (parts of this figure were obtained from https://smart.servier.com/, accessed on 10 December 2024).

Given these risks, RA patients require specialized monitoring to prevent thromboembolic events, with early diagnosis and proper administration of medication being crucial for patient outcomes [37]. The thrombotic tendency in RA and its inflammatory link to VTE are driven by multiple mechanistic factors, including hypercoagulability, endothelial cell injury, and venous stasis [19,38]. The primary objective of this study is to investigate the pathogenesis of RA, identify contributing factors, explore its inflammatory association with thrombosis, and elucidate how these conditions interact within a shared pathological framework. Additionally, this study reviews current and emerging treatment approaches that may alleviate symptoms of both RA and associated thromboembolic events (Figure 1).

## 2. Materials and Methods

The Scopus database was used to conduct an extensive literature review. The following keywords were used: “rheumatoid arthritis”, “arthritis”, “osteoarthritis”, “inflammatory arthritis”, “autoimmune diseases”, “inflammation”, “thrombosis”, “deep vein thrombosis”, “thromboembolic disease”, “venous thromboembolic events”, “von Willebrand factor”, “genetic”, “epigenetic”, “clot formation”, ”venous clots”, “blood cells”, “platelets”, “anticoagulants”, “antirheumatic drugs”, “antiplatelet drugs”, “anti-inflammatory drugs”, ”DMARDs”, ”biologic DMARDs”, ”targeted synthetic DMARDs”, ”conventional synthetic DMARDs”, ”NSAIDs”, “antithrombotic drugs”, ”joint damage”, “interstitial lung disease”, “vasculitis”, “cardiovascular diseases”, “autoantibodies”, “rheumatoid factor”, “anti-citrullinated protein antibodies”, “venous thromboembolic events”, “immunity”, “anti-inflammatory”, “cardio-protective”, “hypercoagulability”, “endothelial cell injury”, “venous stasis”, ”treatment”, ”PAF”, ”thrombin”, ”ADP”, ”TxA_2_”, ”JAK inhibitors”, ”TNF-α inhibitors”, “interleukin inhibitors”, ”vitamin K”, ”PUFAs”, ”heparin”, and “pharmaceuticals”. These keywords were combined using AND and/or OR operators to refine search queries across multiple databases, including Scopus, Web of Science, PubMed, ScienceDirect, ResearchGate, and Google Scholar.

The literature search was conducted between May and November 2024, focusing on studies published within the last five to seven years. The selection criteria were defined based on metadata retrieved from the mentioned databases. Eligible studies met the following inclusion criteria: (i) exclusively peer-reviewed research articles, (ii) published in English, and (iii) dated between 2018 and 2024. Each selected article was evaluated based on its title, abstract, and keywords, ensuring relevance and quality. Duplicates and unrelated sources were excluded, along with conference papers, books, short surveys, and outdated reviews. However, a few key articles published before 2018 were included as they contained critical information not addressed in more recent publications. The final selection of articles was made collectively by all authors based on their relevance, quality, and alignment with the scope of this study.

## 3. Brief Thrombosis Concept, Risk Factors, Mechanisms, and Treatment Approaches

### 3.1. General Profile of Thrombosis

Thrombosis, whether venous or arterial, is a common condition affecting individuals of all ages, characterized by the formation of a blood clot within a blood vessel. The complications of thrombosis often arise in conjunction with underlying medical conditions, particularly autoimmune diseases like RA. Obstruction of blood vessels by clots can result in partial or complete blockage of arterial, venous, or smaller capillary blood flow, leading to severe clinical consequences [27,39,40,41]. Moreover, venous thrombi form under conditions of low flow and low shear stress, as well as in the presence of hypercoagulability, endothelial cell injury, or vessel wall damage, as initially described by Virchow in 1856 [42]. The scientific community has widely accepted these mechanisms and continues to elucidate the pathological basis of thrombosis [16,30,39,41].

### 3.2. Introduction of Thrombosis Concepts: Deep Vein Thrombosis

**Figure 2:**
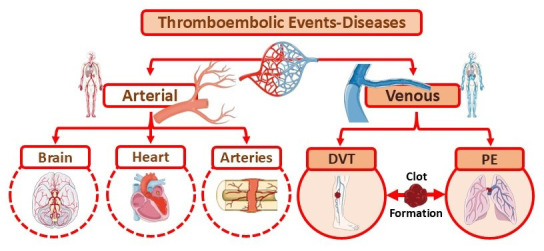
Arterial and venous thromboembolic disorders (parts of this figure were obtained from https://smart.servier.com/, accessed on 11 December 2024).

DVT refers to the formation of a thrombus within deep veins, as opposed to superficial veins [42,43,44]. Thromboembolism involves both an in situ thrombus and an embolus (a dislodged clot) that travels through the circulatory system. Pulmonary embolism (PE) occurs when a clot migrates to the lungs, causing significant morbidity and mortality [45]. The interplay between DVT and PE has led to their classification under the umbrella term venous thromboembolism (VTE), also known as thromboembolic disease. VTE encompasses both arterial (brain, heart, and major arteries) and venous (DVT and PE) thrombotic disorders [45,46,47,48]. Additionally, VTE can affect nearly any blood vessel, influenced by interactions between acquired or inherited thrombophilic predispositions, as well as environmental, genetic, and epigenetic factors (Figure 2) [19,30,45,46,47,48,49,50,51].

### 3.3. Deep Vein Thrombosis and Risk Factors

A key factor contributing to DVT development is surgical intervention, particularly orthopedic, vascular, or neurosurgical procedures, which carry an increased risk of thromboembolic complications, including PE [52]. The reported PE incidence ranges from 0.9–28% for hip arthroplasty and 1.5–10% for knee arthroplasty. Additional risk factors include advanced age, obesity, prior thrombotic events, and malignancy, all of which heighten post-operative thrombosis risk [44,47,48,52,53]. Both prolonged immobilization and surgical interventions serve as major contributors to clot formation and DVT onset, with hip arthroplasty procedures carrying an associated 1.9% risk of heart attack [52,54].

Acute medical conditions like myocardial infarction, acute heart or respiratory failure, and infections further increase the risk of thrombosis [22,55]. While lupus anticoagulants confer a tenfold risk of both first-time and recurrent thrombosis, the relationship between anticardiolipin antibodies and DVT remains scientifically inconclusive [41,56,57]. Other significant risk factors include smoking, atrial fibrillation, chemotherapy, congenital heart disease, sickle cell anemia, thrombophilia, and obesity, all of which must be carefully considered in thrombosis risk assessment [41,56,57].

### 3.4. Molecular Mechanisms of Thrombosis and Thrombo-Inflammation in Thromboembolism

Endothelial injury, whether induced by hypertension, inflammation, endotoxins, reactive oxygen species (ROS), or hypofibrinolysis, is a critical contributor to the majority of thrombotic mechanisms [19,39,41,58]. Hypofibrinolysis is a condition in which the body’s ability to break down blood clots is impaired, leading to prolonged clot persistence and increased risk of vascular complications. In thromboembolism, hypofibrinolysis leads to persistent fibrin deposition, which promotes endothelial dysfunction and vascular occlusion, ultimately resulting in endothelial cell damage and an increased risk of thrombosis [59,60,61].

The onset of arterial thrombosis is often triggered by the rupture of atherosclerotic plaques [62], while microvascular clotting (in arterioles, capillaries, and venules) can lead to extensive organ damage [41,63,64]. Platelet activation and coagulation pathways operate concurrently during thrombus formation, though their exact synergy remains incompletely understood. Platelet activation via collagen and glycoprotein VI (GPVI), as well as coagulation activation through tissue factor (TF)/thrombin, factor VII (FVII), and protein-activated receptors 1 and 4 (PAR1/4), are crucial steps in thrombogenesis. Additionally, platelet activation via PAR1/4 and integrin IIb3 leads to clot stabilization. Both the von Willebrand factor (VWF) and PAR1/4 have been implicated in RA pathogenesis, linking inflammation to thrombosis. Elevated VWF levels correlate with endothelial activation, while PAR1/4 receptors amplify inflammation and pro-thrombotic signaling [65,66].

VWF plays a crucial role in thrombosis by mediating platelet adhesion via the glycoprotein Ib (GPIb) receptor, facilitating platelet aggregation at sites of vascular injury, and promoting the binding of procoagulant factor VIII (FVIII). Nevertheless, some aspects of VWF’s structure, function, and regulatory mechanisms remain poorly understood [65,67,68]. The three primary functional regions of VFW include the following: A1 domain: binds platelet glycoprotein Ibα (GPIbα) receptor, enabling VWF adhesion to collagen; C1 domain: contains the tripeptide Arg-Gly-Asp (RGD) sequence, recognized by β3 integrins (αIIbβ3 and αvβ3); and D′-D3 domain: serves as the binding region for FVIII [65,67,68,69,70,71,72]. VWF size is regulated by ADAMTS13 metalloproteinase, which cleaves the Tyr1605-Met1606 peptide bond explicitly, thereby limiting the thrombotic potential. Larger VWF multimers display increased thrombogenicity, as their multiple binding sites facilitate strong platelet adhesion and clot formation. Controlled VWF release during tissue injury ensures efficient clotting without excessive thrombus formation [65,67,68,69,70,71,72,73].

**Figure 3:**
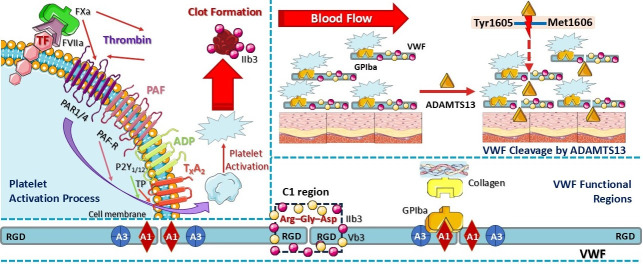
Molecular mechanisms involved in thromboembolism (parts of this figure were obtained from https://smart.servier.com/, accessed on December 11 2024).

Beyond VWF and thrombin, platelet agonists such as adenosine diphosphate (ADP), platelet-activating factor (PAF), and thromboxane A2 (TXA2) contribute to the prolonged activation and aggregation of platelets. Chronic inflammatory conditions, including RA, may enhance several thrombotic cascades, increasing the risk of atherosclerotic, rheumatic, and atherothrombotic events. VWF, thrombin, PAF, ADP, and T_X_A_2_ collectively induce platelet activation and leukocyte recruitment, including neutrophils, monocytes, dendritic cells (DCs), T cells, and B cells. This process is mediated via adhesion proteins, chemokines, and receptor interactions, including PAR1/4 (for thrombin activation), PAF receptor (PAF-R, for PAF), purinergic P2 receptor (P2Y_1_ and P2Y_12_, for ADP signaling), and thromboxane prostanoid receptor (T_X_A_2_ for T_X_A_2_ binding) (Figure 3) [62]. Moreover, pro-inflammatory mediators like tumor necrosis factor alpha (TNF-α) and interleukins (ILs) can exacerbate the inflammatory–thrombotic–coagulation crosstalk, further intensifying thrombo-inflammatory responses [62].

### 3.5. Blood Cells and Role of Platelets in Thrombosis

Endothelial cells form a specialized thin-layered epithelial tissue that lines the inner surface of blood vessels, acting as a barrier between the vessel wall and circulating blood. In the context of blood clotting, endothelial cells play a crucial role in regulating platelet signaling and coagulation [74,75,76]. Interestingly, platelets interact with the vessel wall, detecting environmental changes and relaying signals to other cells. Through specific receptors, platelets mediate vascular homeostasis and regulate thrombosis by controlling the release of granules, RNA transport, and mitochondrial secretion. Platelets maintain vascular homeostasis by specifically secreting mitochondrial components, including mtDNA, adenosine triphosphate (ATP), and ROS, which regulate coagulation, inflammation, and endothelial repair. These factors help balance thrombosis and fibrinolysis while supporting endothelial function and immune responses [76,77,78,79].

**Figure 4:**
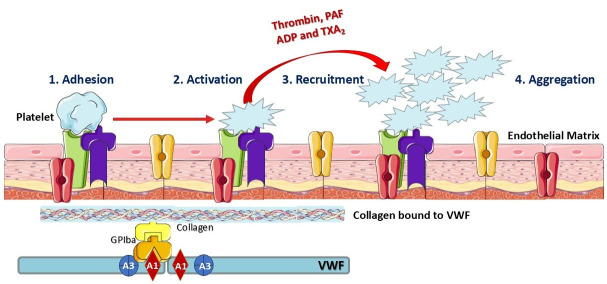
Role of platelets in thrombosis (parts of this figure were obtained from https://smart.servier.com/, accessed on 11 December 2024).

Endothelial cells maintain a balance between procoagulant and anticoagulant mechanisms. Under normal conditions, they form a non-adherent surface that prevents platelet activation and coagulation. However, at sites of vascular injury, when a blood vessel is damaged, platelets adhere to the exposed extracellular matrix. This initiates platelet–platelet interactions that may lead to thrombus formation. This clotting response seals the injured vessel, preventing excessive blood loss. Platelet adhesion triggers the activation and subsequent release of key prothrombotic factors, including ADP, T_X_A_2_, PAF, and thrombin. These factors stimulate further platelet recruitment and aggregation, leading to clot formation. Hence, endothelial cells serve as a structural and functional platform for the formation of procoagulant complexes and the regulation of coagulation [27,65,76,77,78,80]. The general role of platelets in thrombosis is illustrated in Figure 4.

### 3.6. Brief Introduction to Antiplatelet and Anticoagulant Drugs in the Treatment of Thrombosis Incidence Linked to Rheumatoid Arthritis

Fixed-dose, weight-adjusted, subcutaneous low-molecular-weight (LMW) heparin is reported to be at least as effective and safe as intravenously administered unfractionated heparin [81]. It is considered superior due to its more predictable dose–response relationship, longer half-life, and lower risk of immune-mediated thrombocytopenia or osteoporosis. When administered once daily at a dose of 150–200 U/kg, it demonstrates similar efficacy and safety to a twice-daily regimen of 100 U/kg of anti-factor Xa. For long-term protection against thrombus progression and recurrence, vitamin K antagonists can be used in conjunction with heparin following a diagnosis of DVT [81,82,83].

Arterial clots form under high-shear conditions, resulting in platelet-rich thrombi. In contrast, venous thrombi—which develop under low-shear conditions—are rich in trapped red blood cells but contain fewer platelets [84]. Antithrombotic therapy comprises antiplatelet, anticoagulant, and fibrinolytic agents, which effectively target both arterial and venous thrombi. However, due to the high platelet content of arterial thrombi, antiplatelet therapy is primarily used for the prevention and treatment of thrombosis. Conversely, anticoagulants are particularly effective for conditions like atrial fibrillation. Notably, antiplatelet drugs are less effective than anticoagulants in reducing the risk of VTE [31,56,81,82,83,85].

#### 3.6.1. Action of Antiplatelet Drugs

In the vascular system, platelets are maintained in an inactive state by the release of nitric oxide (NO) and prostacyclin from endothelial cells. These cells also express adenosine diphosphatase (ADPase), which degrades extracellular ADP, thereby preventing platelet activation. During vascular wall damage, the release of these antiplatelet substances is restricted, resulting in exposure of the subendothelial matrix. Platelets adhere to exposed collagen and VWF factor via specific receptors, integrating signals from these interactions to alter their shape, secrete ADP from dense granules, and synthesize T_X_A_2_. These substances act as platelet agonists, amplifying the aggregation response. Vessel wall disruption also exposes blood to cells expressing TF, initiating clot formation by triggering the exposure of platelet surface phosphatidylserine [31,85,86]. Coagulation factor complexes assemble then on the platelet anionic surface, leading to thrombin generation. Subsequently, the platelet receptor GPIIb/IIIa (αIIbβ3) enhances fibrinogen binding, facilitating platelet–platelet interactions and the formation of platelet aggregates. Thrombin-derived fibrin strands interweave these aggregates, ultimately forming a platelet–fibrin plexus that stabilizes the clot [31,85,86].

Antiplatelet agents can be categorized by their mechanism of action, targeting platelet adhesion, activation, aggregation, and inflammation-associated thrombosis. Drugs such as aspirin, clopidogrel, dipyridamole, and cilostazol inhibit platelet activation, while GPIIb/IIIa antagonists block platelet aggregation [33,87,88,89,90]. Aspirin (acetylsalicylic acid), a non-steroidal anti-inflammatory drug (NSAID), irreversibly inhibits cyclooxygenase (COX-1) by acetylating a serine residue, thereby reducing prostaglandin H_2_ and T_X_A_2_ biosynthesis. This impairs platelet aggregation without directly affecting ADP or thrombin-mediated activation. Dual or triple therapy combining aspirin with a P2Y_12_ inhibitor or an oral anticoagulant is considered a highly effective approach [91,92,93].

Beyond COX-1 inhibition, aspirin modulates interleukin-4 (IL-4) and nuclear factor kappa B (NF-κB) gene expression via non-COX-dependent pathways, exerting antioxidant and estrogen-modulatory effects [62,94]. It also suppresses TNF-α-induced matrix metallopeptidase-9 (MMP-9) expression via the NF-κΒ and p38 mitogen-activated protein kinase (MAPK) pathways, either alone or in combination with clopidogrel, reducing thrombosis markers such as TF, C-reactive protein (CRP), TNF-α, p-selectin, and cluster of differentiation 40 (CD40L). In RA, NSAIDs such as aspirin are first-line treatments aimed at reducing pain and inflammation in tender and swollen joints [62,94,95].

In RA patients at risk of CVD development, aspirin therapy offers multiple benefits for early CVD prevention. However, long-term use may lead to adverse effects, including severe bleeding and gastrointestinal (GI) complications. Low-dose aspirin combined with omega-3 (ω-3) polyunsaturated fatty acids (PUFAs) or placebo has been associated with decreased vascular event risk in RA patients but also with a higher bleeding risk [62]. A recent randomized clinical trial suggested that salicin, a natural precursor to aspirin derived from willow bark, reduced pain and improved overall health in patients with RA. However, such findings require further validation in more extensive trials [95].

Several second-line antiplatelet therapies have displayed potency in RA and thrombosis management. Clopidogrel, a P2Y_12_ receptor inhibitor, is commonly used to prevent platelet activation. In contrast, PAR antagonists, such as atopaxar and vorapaxar, target thrombin-mediated pathways, as well as the COX and ADP cascades, and they are gaining interest as novel therapies [93,96,97]. Joint PARs (PAR-1, PAR-2, and PAR-4) play a role in pain, inflammation, and vascular reactivity in arthritis [98]. PAR-1 antagonists, in conjunction with aspirin or P2Y_12_ inhibitors, have demonstrated potent antithrombotic activity [62,98,99,100,101]. Moreover, PAR-1 antagonists also exhibit anti-nociceptive properties, reducing inflammatory pain responses. PAR-2 and PAR-4 modulate joint inflammation and leukocyte recruitment, acting as pro-nociceptive and pro-inflammatory mediators. Although clinical trials have shown promising results, the long-term efficacy and safety of PAR antagonists and P2Y_1_/_12_ inhibitors require further investigation [98].

Natural compounds integrated into nutraceuticals and functional food supplements are emerging as safe and effective alternatives for long-term thrombosis management [62]. Fruitflow^©^, a tomato-derived antiplatelet nutraceutical, has been shown to suppress platelet activity and T_X_A_2_ generation more effectively than aspirin [102]. It reduces platelet hyperactivity, granule secretion, and thrombin generation [103,104].

Fish oil supplements, rich in ω-3 (and ω-6) PUFAs, particularly docosahexaenoic acid (DHA) and eicosapentaenoic acid (EPA), exhibit strong anti-inflammatory and antithrombotic properties [27,62,105,106,107,108,109]. The beneficial effects of these natural supplements, primarily attributed to their high concentrations of DHA and EPA, include the modulation of platelet activity and reduction in thrombin generation. These effects are mediated through the regulation of pro-inflammatory transcription factors, alteration of cell membrane phospholipids, disruption of lipid rafts, and binding to specific G-protein-coupled receptors [62]. Furthermore, ω-3 PUFAs have been shown to lower the post-operative risk of DVT and PE, particularly in elderly patients with proximal femoral fractures [105,106,107]. Additionally, combinations of aspirin with DHA or EPA and ticagrelor with DHA have been associated with reduced arachidonic acid (AA)-initiated platelet aggregation. Similarly, vorapaxar combined with EPA exhibited a synergistic inhibitory effect on thrombin receptor activator for peptide 6 (TRAP-6)-induced platelet aggregation and procaspase-activating compound 1 (PAC-1) binding [108].

Lipid mediators derived from ω-3 PUFAs, including resolvins, protectins, maresins, and lipoxins, function alongside aspirin and are collectively classified as specialized pro-resolving mediators (SPMs) [62,109,110,111,112,113,114,115]. SPMs are biosynthesized during the resolution phase of inflammation, where a shift occurs from the production of pro-inflammatory mediators (such as leukotrienes and prostaglandins) to pro-resolving lipid mediators. Resolvins are synthesized from EPA and DHA via COX, lipoxygenase, and cytochrome P450 oxidases, leading to a cascade of anti-inflammatory and pro-resolving effects [62,109,110,111,112,113,114,115]. SPMs function by inducing the resolution of acute and systemic inflammation, terminating leukocyte infiltration, regulating pro-inflammatory markers, and enhancing neutrophil clearance and debris removal by macrophages [62].

Resolvins E1 and E2, derived from EPA, play a key role in inflammation resolution by reducing human platelet polymorphonuclear neutrophil (PMN) aggregation, enhancing non-phagocytic macrophage activity, increasing IL-10 levels, and downregulating leukocyte integrin activation and responses to PAF, ADP, and T_X_A_2_ [62,110]. Similarly, protectins and programmed cell death protein 1 (PD1), generated by DHA, exhibit anti-inflammatory effects and are upregulated following a myocardial infarction [110,116]. Additionally, maresins, synthesized by DHA, have been linked to macrophage-dependent cardiac tissue regeneration by enhancing transforming growth factor-beta (TGF-β) secretion, promoting phagocytosis, inhibiting NF-κB signaling, and reducing pro-inflammatory mediators, such as IL-6 and TNF-α [110,117]. Lastly, lipoxins, which are synthesized via lipoxygenases from AA, have been shown to inhibit neutrophil oxidative burst and exhibit significant anti-thrombotic action [110,118]. Despite the promising therapeutic potential of nutraceuticals, further clinical trials are necessary to validate their safety, efficacy, and long-term preventive effects in managing thrombosis and inflammation [62].

#### 3.6.2. Action of Anticoagulant Drugs

Warfarin is a widely used anticoagulant; however, the scientific community has shifted its focus toward the development of oral drugs targeting thrombin (factor IIα) or factor Xa, both of which play key roles in the coagulation cascade [56]. Thrombin is the most potent platelet agonist, coordinating platelet activation, aggregation, and coagulation. Due to its multiple functions in clot formation, thrombin inhibition not only prevents fibrin formation but also attenuates platelet activation [119]. The first oral thrombin inhibitor tested was ximelagatran, but it was later discontinued due to hepatotoxicity [120].

In the case of factor Xa, its binding to factor Va on activated platelets leads to the formation of the prothrombinase complex, a potent activator of prothrombin. Both thrombin and factor Xa inhibitors offer a significant advantage over traditional anticoagulants, as they are administered in fixed doses, do not require routine coagulation monitoring, are unaffected by any food components, and have a lower potential for drug interactions. By simplifying anticoagulation therapy, these agents have the potential to improve long-term antithrombotic therapy treatment [56,57,121,122,123,124].

Direct oral anticoagulants (DOACs), including dabigatran, rivaroxaban, and apixaban, have been approved over the years and are widely used to manage thrombotic disorders [32,125,126]. While DOACs are not explicitly indicated for treating RA, they play a critical role in managing thrombosis risk in RA patients, particularly for conditions such as VTE. Chronic inflammation observed in RA cases increases VTE risk, and the decision to use DOACs as the treatment option should be individualized [127]. More specifically, DOACs, which are also classified as non-vitamin K oral anticoagulants (NOACs), offer several advantages over traditional anticoagulants such as warfarin and heparin [32,125,126]. Dabigatran is a direct thrombin (factor IIα) inhibitor that prevents fibrin formation [56,128], whereas rivaroxaban and apixaban are factor Xa inhibitors, which block critical coagulation enzymes, thereby reducing thrombin production [56,129,130,131]. DOACs are used for the prevention and treatment of various conditions, including VTE, DVT, PE, cerebral sinus vein thrombosis, thrombocytopenia, thrombophilia, stroke prevention in non-valvular atrial fibrillation [132], and post-operative prophylaxis of DVT after surgery [133].

A preclinical animal study found that dabigatran’s thrombin inhibition led to the suppression of the kallikrein–kinin system (KKS) and the expression of toll-like receptor 4 (TLR4). This was associated with reduced receptor activator of nuclear factor-kappa B (RANKL) and NO levels, suggesting that dabigatran exhibits antioxidant, anti-inflammatory, and potential antirheumatic effects [134]. The safety and efficacy of DOACs in preventing thromboembolic events such as VTE, stroke, and bleeding complications have been confirmed in major clinical trials, including RE-LY (dabigatran), ROCKET-AF (rivaroxaban), and ARISTOTLE (apixaban). These studies have shown that DOACs are at least as effective as warfarin or heparin while offering several key benefits [125,135,136]. DOACs over traditional anticoagulants require fixed dosing with minimal monitoring and display a rapid onset of action, fewer drug interactions, a 22% reduction in ischemic stroke and VTE risk, and a 17% lower risk of significant bleeding. Despite these advantages, DOACs have several limitations, including bleeding risk, limited availability of specific antidotes, and contraindications in RA patients with comorbidities [136,137,138].

## 4. Rheumatoid Arthritis Epidemiology, Mechanisms and Complications, Thrombosis Connection, and Treatment Approaches

### 4.1. General Profile of Rheumatoid Arthritis

Rheumatoid arthritis (RA) is a chronic autoimmune disease characterized by persistent systemic inflammation and an unknown formal etiology. It causes irreversible joint damage and is correlated with extra-articular manifestations like ILD, vasculitis, CVDs, and ocular involvement [1,35,36,139]. Multiple types of arthritis have been investigated and classified into non-inflammatory arthritis (like osteoarthritis) and inflammatory arthritis, which may be triggered by crystal deposition disorders, bacterial or viral infections (such as *Staphylococcus aureus*, *Neisseria gonorrhea*, Lyme disease complications, Parvovirus, Enterovirus, and Hepatitis B (HBV) and E (HEV)), or autoimmune processes. RA belongs to a heterogeneous group of rheumatic diseases, which also includes systemic lupus erythematosus (SLE), Sjögren’s syndrome, systemic scleroderma, spondylarthritis (SpA), psoriatic arthritis, vasculitis, sarcoidosis, and idiopathic inflammatory myopathies (IIM) such as inflammatory myositis. Since many rheumatic diseases share similar clinical presentations, including swollen and tender joints, differential diagnosis is crucial for determining appropriate treatment establishment [1,17,35,36,139,140]. Beyond its significant morbidity, RA is also linked to reduced life expectancy, primarily due to premature cardiovascular mortality. Current research has focused on arterial atherosclerotic disorders, including increased myocardial infarction and ischemic stroke [2,141].

Medical advancements in diagnosis and systemic treatment have significantly reduced disease activity and prevented systemic complications [9,47]. Early diagnosis is crucial to treatment success, particularly in high-risk populations. Modern treatment strategies involve measuring disease activity using complex biomarkers, the development of DMARDs, and biological and targeted synthetic drugs such as Janus kinase (JAK) inhibitors [17,37,47,142,143,144]. Furthermore, ongoing drug development efforts have led to the emergence of therapies with improved efficacy and safety profiles. However, further research is necessary to optimize treatment outcomes [17,37,38,47,142,143,144].

#### Epidemiology of Rheumatoid Arthritis

Scientists estimate that approximately 50% of the risk of developing RA is attributed solely to genetic and epigenetic factors. RA affects around 0.5–1% of adults in developed countries and is three times more common in women than men. The prevalence of RA increases with age, primarily affecting women over 65 years old, suggesting that hormonal factors may play a significant role in its pathogenesis [1,35,139]. However, recent studies indicate that RA incidence peaks at an earlier age—around 55—due to menopause incidence [145]. Interestingly, RA occurs more frequently in Northern Europe and North America compared to developing world areas such as West Africa, highlighting potential geographic and environmental influences on disease prevalence [17].

### 4.2. Mechanisms and Complications of Rheumatoid Arthritis

A leading hypothesis on RA pathogenesis suggests that dysregulated citrullination leads to the production of ACPA antibodies. RA progression is variable, often characterized by episodic flares that—in the absence of optimal treatment—gradually worsen and result in irreversible joint damage [1,15,35,139,146]. RA may develop at target sites due to aberrant self-protein citrullination, triggering the formation of autoantibodies against citrullinated peptides. Several genetic and epigenetic factors contribute to the onset of RA, including gene mutations (e.g., *HLA-DRB1*), epigenetic alterations (e.g., DNA methylation, histone modifications, and post-translational modifications), and environmental triggers (e.g., poor dietary habits, smoking, sedentary lifestyle, and infectious agents) [147].

RA is classified into two main subtypes based on ACPA presence: ACPA-positive RA, which exhibits a more pronounced clinical phenotype, and ACPA-negative RA, with distinct genetic patterns and immune responses to citrullinated antigens [146,147,148,149,150]. Citrullination is catalyzed by peptidyl arginine deiminase (PAD), a calcium-dependent enzyme that converts positively charged arginine into a polar but neutral citrulline residue via post-translational modification. In RA, PAD enzymes can be secreted by granulocytes and macrophages, amplifying immune responses [151]. ACPAs are detected in approximately 67% of RA patients, serving as a precise (>97%) diagnostic marker for early, undifferentiated arthritis and an indicator of disease progression [146,147,148,149,150].

**Figure 5:**
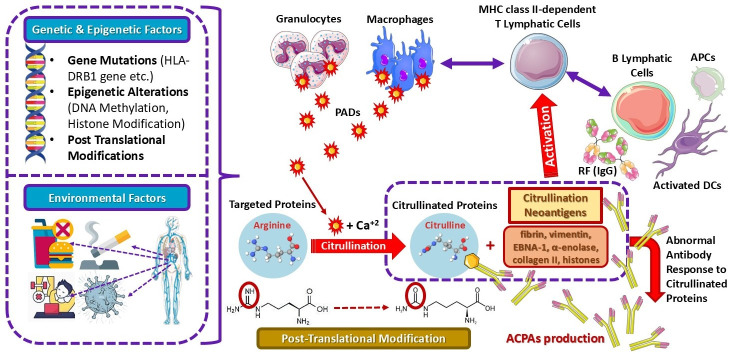
Mechanisms and risk factors of rheumatoid arthritis (parts of this figure were obtained from https://smart.servier.com/, accessed on 10 December 2024).

Several citrullinated proteins have been identified as ACPA targets, including fibrin, vimentin, Epstein–Barr nuclear antigen 1 (EBNA-1), α-enolase, collagen type II, and histones [15,139,147,152,153]. The most potent genetic risk factor for ACPA-positive RA is associated with genes encoding human leukocyte antigen-DR isotype (*HLA-DR*), particularly *HLA-DR1* and *HLA-DR4*, collectively known as shared epitopes (SEs). Additionally, the tyrosine phosphatase non-degrading protein type 22 (PTPN22) has been implicated in ACPA-positive RA due to polymorphisms that inhibit T lymphocyte activation, ultimately promoting ACPA production [154]. An increased type I interferon gene (IFN) response plays a critical role in Th1 cell differentiation and B cell proliferation, thereby further driving ACPA generation. As a consequence, citrullinated neoantigens activate major histocompatibility complex (MHC) class-II-dependent T cells, which, in turn, assist B cells in amplifying ACPA production, leading to a loss of immune tolerance (Figure 5) [15,147,155,156].

Environmental triggers also play a crucial role in the generation of ACPAs. ACPAs can be detected long before joint symptoms appear, suggesting that the joints may not be the initial trigger for autoimmunity. Lung exposure to environmental pollutants, like smoke, silica dust, silica nanoparticles, and carbon nanomaterials, may stimulate mucosal receptors, calcium (Ca^2+^)-mediated PADs, antigen-presenting cells (APCs), classical DCs, RF, and B cell, promoting citrullination and ACPA production. Smoking, in conjunction with the *HLA-DR* SE gene, has been shown to elicit RA-specific immune responses to citrullinated proteins [38,147,157]. Interestingly, ω-3 fatty acids were able to reduce ACPA production risk and potentially prevent RA development. Studies suggest that ACPA-positive individuals who do not have RA may remain asymptomatic for years, indicating that ACPA presence alone does not necessarily trigger RA onset [38,147,157].

Although hormonal factors have been linked to the pathogenesis of RA, no direct association has been established between hormonal imbalances and the production of ACPAs. Understanding the precise contribution of each risk factor is critical for developing early diagnosis tools and identifying molecular targets for personalized medicine [146]. The overall mechanisms and risk factors associated with RA are depicted in Figure 5.

### 4.3. Complications of Thrombosis Involvement in Rheumatoid Arthritis

**Figure 6:**
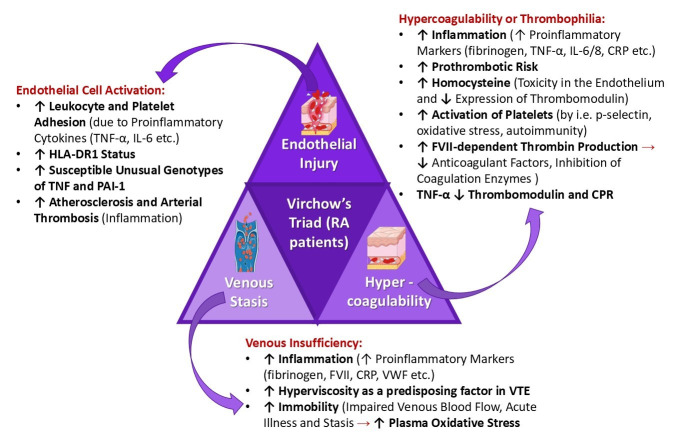
Virchow’s pyramid in patients with rheumatoid arthritis (parts of this figure were obtained from https://smart.servier.com/, accessed on 11 December 2024).

RA is primarily associated with joint pain and physical disability, but CV events, including VTE, largely determine its prognosis. RA patients share an increased risk of arterial or venous thrombosis, as chronic inflammation exacerbates a hypercoagulable state, leading to a higher risk of DVT [147]. Studies suggest that RA patients have twice the risk of developing VTE, with an incidence rate of four cases per 1000 people. This thrombotic tendency in arthritis patients appears to stem from vascular injury, hypercoagulability, and venous stasis—collectively known as the three parts comprising Virchow’s triad—which are highly activated in RA (Figure 6) [28,36].

RA results in a breakdown of peripheral tolerance to autoantigens due to prolonged immune activation and dysfunction of both T and B cells. The presence of ACPAs and immunoglobulin G (IgG) triggers the formation of immune complexes, leading to the overproduction of pro-inflammatory mediators like TNF and IL-6 [38,158,159,160]. Moreover, excessive intra-abdominal fibrin deposition occurs when fibrin clots interact with endothelial cells. More specifically, intra-abdominal fibrin accumulation occurs when persistent inflammation and immune complex formation enhance fibrin clot generation while impairing fibrinolysis. This process is driven by increased fibrinogen levels, thrombin activity, and reduced plasmin-mediated fibrin degradation [161]. While the precise mechanisms underlying the thrombotic risk in RA patients remain unclear, the multifactorial nature of this association has led to the application of Virchow’s triad as a model to describe the prothrombotic state in RA. Systemic inflammation related to RA directly impacts at least two to three components of the triad (Figure 6) [38,158,159,160].

#### 4.3.1. Endothelial Injury

Recent studies suggest that endothelial dysfunction occurs in the early stages of RA as a pre-inflammatory state, promoting increased endothelial cell activation, permeability, leukocyte-platelet adhesion, and monocyte–endothelial cell interactions [38,162,163]. Endothelial dysfunction in RA has been linked to inflammation, *HLA-DR1* status, specific TNF-α genotypes, and elevated levels of plasminogen activator inhibitor-1 (PAI-1). During inflammation, monocytes stimulated by pro-inflammatory cytokines (IL-1β, IL-6, and TNF-α) induce the expression of intercellular adhesion molecules, which contribute to the development of thrombosis. Additionally, coagulation-related endothelial factors, such as VWF and PAI-1, are significantly elevated, reinforcing the RA–thrombosis link [38,162,163,164]. As a result, endothelial dysfunction contributes not only to venous thrombosis but also to arterial thrombosis by accelerating the progression of atherosclerosis. The pathophysiological features of atherosclerosis and VTE share common inflammatory pathways involving endothelial injury, hypercoagulability, and systemic inflammation (Figure 6) [38,162,163].

#### 4.3.2. Hypercoagulability

Inflammation directly influences thrombosis by increasing procoagulant factors, reducing anticoagulants, and suppressing fibrinolysis. Several inflammatory markers (e.g., fibrinogen, IL-6, IL-8, TNF-α, and CRP) are closely linked to prothrombotic activity and the risk of VTE. Evidence suggests that coagulation processes in RA are activated at both extravascular and intravascular sites [38,164,165]. Extravascular activation occurs when chronic inflammation leads to the deposition of fibrin in synovial tissue, promoting pannus formation and joint destruction. In contrast, intravascular activation is driven by systemic inflammation, endothelial dysfunction, and increased circulating procoagulant factors, which contribute to a hypercoagulable state and elevate the risk of thrombotic events, including VTE [38,61,166,167]. In addition, elevated homocysteine levels are frequently observed in RA, which may contribute to endothelial toxicity and reduced thrombomodulin expression, thereby further increasing the risk of thrombosis [38,164,165].

In RA, platelets are activated by inflammatory mediators, such as p-selectin, which contributes to the formation of clots. Moreover, oxidative stress and autoimmunity enhance the release of platelet-derived microparticles, which, in turn, stimulate factor-VII-dependent thrombin production. Inflammation also downregulates anticoagulant factors and delays the inhibition of coagulation enzymes, further sustaining a prothrombotic state. Among pro-inflammatory cytokines, TNF-α plays a key role in reducing thrombomodulin and CRP levels, impairing natural anticoagulation mechanisms (Figure 6) [38,164,165].

#### 4.3.3. Venous Stasis

Currently, there is limited research on the specific mechanistic links between RA and increased plasma viscosity. Factors such as CRP, FVIII, fibrinogen, and VWF are commonly elevated in RA and have been associated with hyperviscosity, which may promote the development of VTE and thrombosis. While direct evidence linking VWF to hyperviscosity in RA is limited, the roles of fibrinogen, factor VII, and CPR in increasing plasma viscosity have been extensively documented. Further studies are required to establish a direct mechanistic link between these factors and RA-related hyperviscosity [61,168,169,170,171,172].

Plasma hyperviscosity, particularly during acute joint inflammation, is considered an important predisposing factor for VTE. Furthermore, prolonged immobility during severe RA flare-ups or critical illness may lead to impaired venous circulation, further contributing to venous stasis and thrombus formation. Oxidative stress associated with acute inflammation in RA contributes to endothelial dysfunction and platelet activation, both of which are key components of Virchow’s triad and may predispose patients to thrombus formation (Figure 6) [35,38,173,174,175].

Although RA is not a direct cause of thrombosis, chronic RA-related inflammation, alterations in prothrombotic blood composition, and endothelial dysfunction may contribute to the development of atherosclerosis. Notably, a high prevalence of lesions in peripheral arteries (19.6%) and veins (7.2%) highlights a potential link between RA and thrombosis [165,176]. In this context, numerous RA patients receiving JAK inhibitors like baricitinib and tofacitinib have developed thromboembolic complications, suggesting an increased risk of VTE [177]. Additionally, RA disease activity has been affiliated with a significantly higher VTE risk, with a risk ratio of 1.88 in similar clinical trials. The use of biologic DMARDs and JAK inhibitors has also been identified as a potential risk factor for VTE, underscoring their relevance in VTE risk stratification [38,178,179]. Moreover, chronic inflammation in RA patients with a history of smoking has been linked to pulmonary thromboembolism and ILD [180]. Epidemiological data further indicate that RA patients face a 48% CVD risk [181]. Mechanistic evidence supporting the association between RA and thrombotic comorbidities includes elevated pro-inflammatory cytokines [165], increased TF expression [182], enhanced fibrin deposition [165], platelet activation during thrombocytosis [183], increased platelet microvesicles [78], or elevated fibrinogen levels [184]. Despite these findings, available clinical data remain insufficient to establish RA as an independent risk factor for thrombosis definitively. Therefore, further large-scale clinical trials are required to validate this hypothesis [165].

### 4.4. Role of Platelet-Activating Factor, Adenosine Diphosphate, Thromboxane A_2_, and Thrombin Mediators Associated with Thrombosis Involvement in Rheumatoid Arthritis

#### 4.4.1. Role of PAF

PAF is a phospholipid inflammatory mediator implicated in numerous chronic, inflammation-related diseases. PAF (mainly in the form of alkyl-PAF), along with PAF-like lipid molecules (PAFLLs), is produced through two distinct enzymatic pathways: the de novo and re-modeling pathways [27,109,185]. These molecules exert their biological effects through a specific G-protein-coupled seven-transmembrane receptor known as the PAF receptor (PAF-R) [27,109]. PAF, PAFLLs, and PAF-Rs participate in many inflammatory cascades affiliated with several chronic disorders, including atherosclerosis [186,187], CVDs [188,189], cancer [27,190], renal disorders [191,192], cerebrovascular and central nervous system (CNS) disorders [193,194], allergic conditions like asthma [27,109], infections [193], and autoimmune diseases like RA [27,109,195,196]. Regarding human platelets, PAF induces shape alterations, aggregation, and granule compound release by triggering the phosphatidylinositol cycle, intracellular Ca^+2^ mobilization, and AA release. Furthermore, PAF and PAF-Rs facilitate the uptake of oxidized low-density lipoproteins (ox-LDLs), which comprise PAF-like oxidized phospholipids, further contributing to both inflammatory and atherogenic processes [62].

Dysregulated PAF metabolism leads to elevated PAF levels, exacerbating inflammation and contributing to RA progression. This process is associated with endothelial dysfunction, platelet and leukocyte hyperreactivity, and increased oxidative and nitrosative stress, mediated by ROS and nitric oxide synthase (NOS) activity, respectively. PAF stimulates the production of pro-inflammatory cytokines, chemokines, and lipid mediators, further intensifying cascades in RA and other diseases [27,62,174,175]. Beyond RA’s onset, PAF also drives disease progression by affecting macrophages and endothelial and smooth muscle cells. PAF activity is regulated by PAF acetyl-hydrolases (PAF-AHs), which degrade PAF and are also modulated by TNF-α-dependent molecular cascades. Since TNF-α antagonists have been shown to inhibit platelet activation in RA patients, targeting PAF and its receptor represents a promising therapeutic strategy. Reportedly, RA patients exhibit elevated circulating platelet activity, which is associated with leukocyte activation and the development of inflammatory manifestations in the vessels and synovium [62]. PAF generally contributes to endothelial dysfunction, enhances vascular permeability, promotes foam cell formation, facilitates atherogenesis, promotes thrombus formation, and is associated with vasodilation-related inflammatory pain. These effects exacerbate joint inflammation and damage in RA patients [27,109,195,196].

Given its crucial role in RA pathogenesis, targeting PAF and PAF-R presents a novel therapeutic approach. Anti-PAF and anti-PAF-R drugs could serve as potential RA treatments [27,109,173,195,196]. Additionally, recent studies have highlighted the therapeutic potential of nature-derived polar lipids, which inhibit the PAF/PAF-R pathway and regulate PAF metabolism with no reported side effects. Such bioactive lipids exhibit anti-thrombotic, anti-inflammatory, and antirheumatic properties, paving the way for novel pharmaceutical interventions [109,185,197,198].

#### 4.4.2. Role of Thrombin

Thrombin interacts with platelets through two G-protein-coupled receptors, PAR1 and PAR4. The generation of thrombin occurs in three key stages: the initiation stage, where prothrombin is cleaved by FVII upon tissue injury, leading to the formation of α-thrombin; the propagation stage, where α-thrombin is further cleaved to β-thrombin; and the amplification stage, where β-thrombin undergoes hydrolysis to γ-thrombin [119,199]. All thrombin isoforms can induce platelet activation via PAR receptors as well as interact with CRP, promoting inflammatory responses and thrombosis [62]. At sites of vascular injury, thrombin plays a crucial role in thrombo-inflammation by regulating leukocyte activity, increasing endothelial permeability, enhancing platelet adhesion, stimulating the release of pro-inflammatory cytokines, and promoting the production of PAF. RA has been strongly associated with increased thrombotic activity, as evidenced by elevated levels of thrombin markers, including thrombin–antithrombin complexes, prothrombin fragments, fibrinogen, VWF, TF, D-dimer, and impaired fibrinolysis [38,99,121,200,201]. A clinical study by Undas et al. [119] reported that RA patients exhibit elevated factor VIII levels, along with increased TF pathway inhibitor (TFPI) levels, which can partially counteract the prothrombotic effects of FVIII. Another study found that thrombin formation is significantly increased in RA patients, although paradoxically, some reports indicate a decrease in thrombin-generating capability in certain RA cases [202].

Interestingly, platelets derived from RA patients demonstrate impaired responsiveness to thrombin stimulation. They fail to adequately induce α-granule secretion and exhibit reduced activation of integrin αIIb3, which is associated with diminished p-selectin expression and reduced fibrinogen-binding ability. As a result, systemic immune platelet activation in the bloodstream leads to elevated thrombinemia, accompanied by increased expression of TF, higher levels of procoagulant phospholipids, and release of platelet-derived microvesicles [165]. This platelet exhaustion, combined with persistent thrombin generation, contributes to energy depletion, secondary inflammation, and suppression of clot contraction, not only in RA but also in conditions like VTE [98,165]. In cases where thrombosis develops in RA patients, an optimal treatment approach may involve a combination of antithrombotic drugs, antiplatelet agents, anticoagulants, and/or fibrinolytic drugs. This multimodal strategy may help prevent the progression of thrombosis and mitigate RA-associated thrombotic complications [98,165].

#### 4.4.3. Role of ADP

Both ADP and T_X_A_2_ are key contributors to platelet activation and augmentation during hemostasis. ADP acts as a potent agonist of P2 receptors, specifically P2Y_1_, P2Y_12_, and P2X_1_, leading to platelet activation, aggregation, and recruitment. Upon ADP binding to these receptors, the following signaling cascades are triggered: P2Y_1_ and P2Y_12_ receptor activation, intracellular Ca^2+^ increase via phospholipase C and inositol phosphate signaling pathways, as well as inhibition of adenylyl cyclase signaling. The latter leads to reduced intracellular cyclic adenosine monophosphate (cAMP) formation, inhibition of vasodilator-stimulated phosphoprotein phosphorylation, and activation of the glycoprotein IIb/IIIa receptor, promoting platelet aggregation [62,185,203].

ADP further amplifies platelet aggregation by binding to the P2Y_12_ receptor, which activates phospholipase A2 (PLA_2_) through a cytosolic Ca^2+^ increase and stimulates AA metabolism to T_X_A_2_. Alternatively, AA can induce the formation of bioactive lipids via COX-1, leading to the production of prostaglandin G_2_ (PGG_2_) in platelets, the conversion of PGG_2_ to prostaglandin H_2_ (PGH_2_), and the subsequent transformation into T_X_A_2_, which further amplifies platelet activation and aggregation [62]. Several studies have highlighted that blocking P2Y_12_ may have therapeutic benefits in RA and other inflammatory diseases. Specifically, the inhibition of P2Y_12_ with clopidogrel has been demonstrated to reduce osteoclast activity and inhibit age- and tumor-related bone loss, as shown in preclinical animal models [19,203,204].

#### 4.4.4. Role of T_X_A_2_

T_X_A_2_ is a pro-inflammatory and procoagulant factor that promotes excessive platelet activation and recruitment, similar to ADP. T_X_A_2_ diffuses across plasma membranes and binds to specific G-protein-coupled thromboxane prostanoid receptors (TPα and TPβ), which are also expressed in endothelial cells, erythrocytes, and monocytes. Through these interactions, T_X_A_2_ enhances platelet activation, amplifies the effects of other agonists, such as thrombin or collagen, and thus facilitates platelet-endothelium binding, contributing to endothelial dysfunction. Notably, T_X_A_2_ plays a crucial role in RA and synovial cell proliferation, acting via an auto-regulatory feedback loop that sustains inflammation and promotes RA progression [62,205]. T_X_A_2_ is primarily modulated by COX-1 and functions as a potent platelet agonist. Its degradation product, T_X_B_2_, is strongly correlated with RA disease activity, indicating its role in disease severity and progression [84].

Scientific evidence suggests that targeting T_X_A_2_ and/or prostacyclin I_2_ (PGI_2_) receptors may yield cardioprotective and antirheumatic effects [195,196,206]. Additionally, blocking T_X_A_2_ synthase and TP receptor cascades has been proposed as a potential therapeutic strategy. Lipid-metabolite-based drugs have emerged as appealing anti-inflammatory, antirheumatic, and antithrombotic agents. However, further research is required to validate their efficacy and clinical application [62,205].

### 4.5. Role of Anticoagulant, Antiplatelet, and Antithrombotic Therapy in Rheumatoid Arthritis with Thrombosis Incident

In patients with confirmed VTE, rapid initiation of anticoagulant therapy is crucial to prevent clot progression, reduce further complications risk, and lower mortality [207]. Despite RA patients having a higher VTE incidence, long-term systemic anticoagulation is not routinely recommended due to associated risks. However, in high-risk populations, including RA patients undergoing major surgeries such as total knee or hip arthroplasty or those with several thrombotic risk factors, thromboprophylaxis should be considered before VTE occurs to reduce incidence [38,200,208]. Interestingly, even RA patients who have not undergone joint surgery have been found to have twice the risk of VTE compared to non-RA patients [19,38,208]. Hence, prompt anticoagulation lowers the risk of recurrent VTE and mortality within the first few days of therapy initiation [38,209,210].

The most commonly used anticoagulants for VTE prevention and treatment include warfarin and low-MW heparin [31,56,81,82,83,85]. Moreover, antiplatelet agents such as aspirin or clopidogrel have been evaluated for VTE recurrence prevention [33,87,88,89,90]. Nevertheless, conflicting study results indicate that antiplatelet agents may not be as effective as anticoagulants for preventing VTE recurrence. For instance, the EINSTEIN CHOICE trial revealed that aspirin had a limited role in preventing VTE recurrence compared to rivaroxaban, implying that DOACs may offer superior protection. Despite their benefits, anticoagulants have exhibited several adverse effects [56,211].

Current clinical guidelines recommend that after a first VTE episode, patients should receive anticoagulation for at least 3 months; nevertheless, even after discontinuation, the risk of VTE recurrence remains substantial, with a reported 8–10% recurrence rate within the first year. Studies indicate that prolonged anticoagulation beyond the acute phase can reduce VTE recurrence by up to 80% [38]. However, the decision to continue or discontinue anticoagulation therapy must weigh the benefits of lowering VTE recurrence against the increased risk of bleeding [131,211,212,213,214]. For patients without transient (e.g., temporary conditions like surgery or acute illness that increase VTE risk but resolve entirely after recovery) or reversible risk factors (e.g., modifiable conditions like obesity or smoking, which may persist unless actively managed), the risk of VTE recurrence remains at 10% in the first year after stopping the administration of anticoagulation therapy [38].

RA and other chronic inflammatory diseases are recognized as persistent secondary thrombotic risk factors. Based on an individualized assessment of thrombotic and bleeding risk, some RA patients may benefit from either prolonged or indefinite anticoagulation [131,211,212,213,214]. Further studies are therefore needed to establish clear guidelines on the optimal antithrombotic regimen for RA, as no definitive recommendations currently exist regarding the long-term management of VTE in RA [38].

### 4.6. Conventional and Current Treatment Approaches for Rheumatoid Arthritis

#### 4.6.1. Conventional Treatment Approaches

Characterizing risk factors offers valuable insights into the prevention and treatment of RA. The primary goal of RA treatment is to relieve pain, reduce inflammation, and prevent disease progression. First-line management mainly includes NSAIDs and glucocorticoids (GCs), while COX-2 inhibitors are also widely used [209,215]. However, long-term use of NSAIDs and COX-2 inhibitors is associated with increased CVD and thromboembolic risks, whereas aspirin, through its COX-1 inhibition, may have a protective effect against VTE [38].

NSAIDs such as naproxen, ibuprofen, and coxibs are lipophilic compounds that inhibit COX-1 and COX-2, which are upregulated in RA and contribute to inflammation and thrombosis. By decreasing prostanoid synthesis (e.g., prostacyclins, prostaglandins, and T_X_A_2_), NSAIDs provide anti-inflammatory and analgesic effects; however, this inhibition can also lead to several adverse effects [209,215]. COX inhibition can be non-selective (targeting both COX-1 and COX-2) or selective (with either COX-1 or COX-2 inhibitors) [216,217,218]. COX-1 is expressed in most tissues and regulates gastric mucosal protection and platelet aggregation by producing T_X_A_2._ Inhibiting COX-1 reduces platelet aggregation but increases the risk of gastrointestinal irritation and bleeding [219,220,221].

COX-2, an inducible enzyme found in the brain, uterus, and kidneys, is mainly involved in inflammation and pain modulation. COX-2 inhibitors (e.g., celecoxib, rofecoxib, valdecoxib) reduce inflammation with fewer GI side effects (i.e., renal impairment, GI ulcers, GI irritation, and bleeding, and increased CVD risk) than traditional NSAIDs but have been linked to an increased risk of CVD [216,217,218]. Thus, COX-2 inhibitors display multifaceted clinical effects, ranging from reducing pain, inflammation, and colorectal cancer risk to increasing blood pressure and atherothrombotic risk [17,222].

Although COX-2 inhibitors are preferred over conventional NSAIDs due to their lower GI toxicity, they still carry significant CV risks, particularly with long-term or high-dose use. Rofecoxib was withdrawn due to CV risks. Meanwhile, etoricoxib (a selective COX-2 inhibitor) combined with duloxetine has been shown to reduce post-operative pain and opioid use following lumbar laminectomy, especially in RA patients [223]. Celecoxib, commonly used in RA therapy, possesses anti-inflammatory, analgesic (pain-relieving), and antipyretic (fever-reducing) properties and is often co-administered with GCs [224]. However, COX-2 inhibitors have been associated with a 35–42% increased risk of CVD [2,14,225], as well as an elevated risk of arterial hypertension [36] and adverse effects on lipid metabolism due to their interference with cholesterol transport [225]. Furthermore, COX-2 inhibitors may increase the risk of myocardial infarction, stroke, and heart failure [219,220,221]. Interestingly, PGI_2_ produced by COX-2 plays a protective role in the CV system by promoting vasodilation, inhibiting platelet aggregation, and reducing arterial clot formation. Therefore, PGI_2_ lowers the risk of thrombotic events (i.e., heart attacks and strokes), and its inhibition is linked to RA-induced thrombotic events [219,220,221].

Even though systemic GCs are not typically linked to VTE, their use has been shown to increase the risk of thromboembolic events by two to three times, even with short-term administration. This risk is due to GC-induced endothelial injury, which results from primarily decreased NO production that impairs vascular relaxation. Moreover, the increased expression of adhesion molecules that promote leukocyte–platelet interactions and enhanced hypercoagulability, as GCs increase the levels of procoagulant factors (i.e., FII, FVIII, and VWF), has also been associated with a high risk of VTE [19,226,227]. Within five days of GC administration, concentrations of clotting factors and fibrinogen rise, while over time, procoagulant activity could be affected by platelet accumulation inhibition [38,226]. Commonly used GCs include prednisone, hydrocortisone, prednisolone, and dexamethasone, all of which are far more effective than NSAIDs. Nevertheless, long-term use of GCs is associated with notable side effects, such as weight gain, water retention, muscle weakness, and insulin resistance, which often outweigh their benefits, favoring the short-term use of GCs [17,19,228].

Given these risks, the prescription of NSAIDs, COX-2 inhibitors, or GCs in RA patients should always be based on an individualized risk assessment [224]. GC tapering, also known as withdrawal, is crucial to prevent long-term side effects. Explicitly, GCs are often utilized as a “bridge therapy” at the beginning of RA treatment to rapidly reduce inflammation symptoms until DMARDs take full effect, which can take from weeks to several months. Once DMARDs start working, GCs should be tapered off to avoid possible adverse effects (e.g., osteoporosis, hyperglycemia, weight gain) [229,230,231].

Considering the limitations of NSAIDs and GCs, DMARDs have become the second-line treatment option for RA and are classified as conventional, biologic, and targeted synthetic DMARDs. Conventional synthetic DMARDs (csDMARDs) are widely used as a first-line therapy for newly diagnosed RA patients, offering effective disease control with fewer thrombotic risks compared to NSAIDs or GCs [230,232,233]. csDMARDs have not exhibited any safety-related problems regarding VTE and are composed of drugs like methotrexate, leflunomide, hydroxychloroquine, and sulfasalazine [17,38].

Methotrexate remains the gold-standard csDMARD. It is utilized as a long-term therapy for RA, with its high-dose administration affecting coagulation by reducing fibrinogen and coagulation inhibitors (i.e., protein C and antithrombin). It exerts its anti-inflammatory effects—as observed in RA patients—by inhibiting purine biosynthesis, suppressing cytokine production, and enhancing adenosine receptor activity, thereby reducing the thrombotic risk [17,234,235]. Additionally, hydroxychloroquine, an antimalarial medication, exhibits anti-inflammatory and antithrombotic properties, which lower pro-inflammatory cytokine levels. However, high-dose hydroxychloroquine administration has been highly correlated to CVD events like VTE onset and retinal toxicity, leading to pre-retinopathy symptoms, which limits its routine use [17,236,237]. Leflunomide, although practical, is less preferred due to its hepatotoxic effects, which are evident through an increase in liver enzymes; it is reserved for cases of poor tolerance to methotrexate [139]. Similarly, sulfasalazine is also not recommended for long-term use, as its side effects often outweigh its efficacy [17,37].

**Figure 7:**
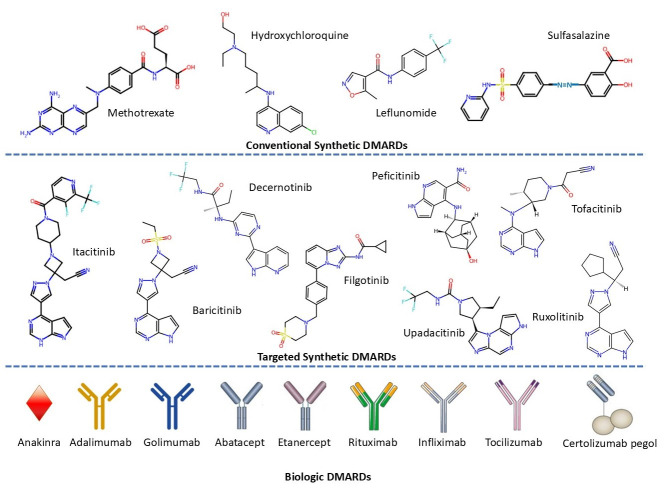
Commonly-used conventional synthetic, targeted synthetic, and biologic DMARDs (molecular structures were obtained from https://molview.org/, accessed on 12 December 2024, while schematic representations of antibodies were obtained from https://smart.servier.com/, accessed on 12 December 2024).

Despite the fact that csDMARDs are widely used [17,38], their poor aqueous solubility, low permeability, and lack of target specificity limit their bioavailability. Moreover, side effects and drug resistance point out the need for more advanced therapeutic interventions. A common RA treatment strategy, especially in cases of moderate-to-severe disease for better control, involves combination therapies with csDMARDs [238]. In a recent study, patients receiving triple csDMARDs (methotrexate, hydroxychloroquine, and sulfasalazine) therapy remained on their treatment longer while attaining low disease activity and remission (6 months after treatment initiation) compared to those on double csDMARDs (methotrexate and leflunomide) therapy (Figure 7) [142,239].

#### 4.6.2. Current and Newly Emerged Treatment Approaches

An approach in RA treatment involves microRNAs (miRNAs), which serve as potent genetic regulators. A single miRNA can influence entire cellular pathways by interacting with a broad spectrum of target genes, making them highly promising therapeutic tools. These molecules can restore altered cell functions in disease phenotypes and regulate key cellular processes, including growth, differentiation, development, and apoptosis. Recent miRNA-based clinical trials have shown promising results in the treatment of RA and cancer [240,241,242]. Additionally, plant-derived miRNAs have emerged as a novel therapeutic avenue, owing to their natural dietary occurrence, minimal side effects, and energy-conserving mechanism of action. These exogenous miRNAs alter signaling by modulating gene expression across species through inter-species or inter-kingdom interactions. This regulatory function enables cells to better cope with complex diseases, and researchers are actively developing miRNA-based therapies that target key pathways involved in RA pathogenesis [240,241,242].

Current RA treatment strategies have significantly evolved to overcome the limitations of csDMARDs, which often exhibit poor bioavailability, reduced efficacy, and tolerance issues. Biologic and targeted synthetic DMARDs are now widely used, either alone or in combination therapy, for superior RA control. Biologic DMARDs (bDMARDs) can be classified, depending on their mechanism of action [17], into TNF-α inhibitors (e.g., etanercept, anti-TNF monoclonal antibodies infliximab, golimumab, adalimumab, certolizumab pegol), B-cell depleters (e.g., rituximab, ofatumumab), B-cell receptor inhibitors (e.g., belimumab, atacicept, tabalumab), antagonists of CD28 on T cells (e.g., abatacept, belatacept), IL-1 inhibitors (e.g., anakinra, canakinumab, rilonacept), IL-6 inhibitors (e.g., tocilizumab, sarilumab, sirukumab, olokizumab, clazakizumab), IL-12/23 inhibitors (e.g., ustekinumab), IL-17 inhibitors (e.g., ixekizumab, secukinumab, brodalumab), granulocyte-macrophage colony-stimulating factor inhibitors (e.g., mavrilimumab, otilimab), and RANKL inhibitors (e.g., denosumab). Since their discovery, these biological agents have transformed RA treatment due to their potent antirheumatic, anti-inflammatory, and even anti-thrombotic properties. However, not all biologics are currently used in routine RA therapy (e.g., IL-1 or IL-12/23 inhibitors), though clinical trials suggest potential future benefits through their systemic use [17,243].

Despite their efficacy, TNF-α inhibitors present dose-dependent risks, including infections, tuberculosis, and lymphoma [173,244,245]. TNF-α is a key pro-inflammatory cytokine in the pathogenesis of RA, driving synovial inflammation and autoimmunity. Blocking TNF-α may help reduce CV risk, myocardial infarction incidence, and VTE onset by reversing inflammatory and thrombotic pathways [246,247]. However, these therapies may induce the production of antiphospholipid antibodies (aPLs), which can paradoxically increase thromboembolism and VTE risk [38]. Innovative delivery methods, such as transdermal microneedle drug delivery and the administration of etanercept or biosimilar TNF-α inhibitors, are being explored as promising alternatives to RA treatment [10,248]. Drugs such as etanercept, infliximab, adalimumab, certolizumab-pegol, and golimumab are anti-TNF-α inhibitors that block the interaction of TNF-α with its receptors and reduce the risk of thromboembolism by controlling systemic inflammation and RA hypercoagulability [249,250]. TNF receptor 2 (TNFR2) neutralization has also been proposed as a novel strategy for developing and regulating IFN-γ and IL-17 production [251].

Cell-based therapies targeting the depletion of B and T cells have also gained traction in the management of autoimmune diseases. B-cell depleters, such as rituximab, and B-cell receptor inhibitors, like atacicept, are a few examples. Additionally, agonists of CD28 on T cells, including abatacept and belatacept, and IL-1/6/12/17/23 inhibitors, like canakinumab, tocilizumab, ustekinumab, secukinumab, and brodalumab, are also available. For instance, rituximab is frequently used in lymphoproliferative disorders and autoimmune diseases, though transient thrombocytopenia has been reported as a side effect [38]. Studies also indicate that combining rituximab with leflunomide enhances the efficacy of RA treatment [252,253]. Furthermore, IL-6 plays a major role in RA pathophysiology, promoting platelet production and the expression of coagulation factors (i.e., TF, FVIII, and VWF) and reducing protein S levels, thereby linking RA to a prothrombotic state [38]. Treating RA patients with IL-6 antagonists in combination with various DMARDs has yielded notable improvements in their symptoms and quality of life [160]. This is because IL-6 inhibitors, such as tocilizumab, have been shown to reduce hypercoagulability markers (i.e., fibrinogen and D-dimer) and improve overall disease outcomes in RA [254,255,256].

Targeted synthetic DMARDs (tsDMARDs) such as JAK inhibitors (i.e., baricitinib, tofacitinib, upadacitinib, peficitinib, filgotinib, decernotinib, ruxolitinib, and itacitinib) are emerging as promising alternatives for RA patients with inadequate csDMARD response. JAKs are cytoplasmic proteins responsible for interconnecting cytokine signaling and the action of signal transducers and activators of transcription (STATs) to control inflammatory and thrombotic responses [17]. JAK inhibitors consequently interfere with cytokine signaling pathways and have displayed higher patient compliance, thus being considered adequate bDMARDs compared to csDMARDs [143] in second-line RA treatment [144]. However, safety concerns remain, as JAK inhibitors have been associated with increased VTE incidence [257], higher rates of severe infections (compared to IL-6 inhibitors) [258], and potential pulmonary and lipid metabolism alterations [259,260,261]. Although challenges persist, further research may enhance JAK inhibitor safety, particularly in younger patients with active refractory RA but without significant CVD risk. Additional clinical trials are necessary before the systematic use of this approach as a first-line treatment is recommended (Figure 7) [262].

#### 4.6.3. Surgery-Related Treatment Approaches

Considering joint surgery in RA patients, treatment approaches reached a peak in the 1990s. Over the past decades, advancements in RA treatment have significantly reduced the need for joint surgery or other surgical interventions, particularly in patients aged 40–59 years, due to early diagnosis and improved medical therapies, including biologic agents [263]. Conversely, patients over 60 years old continue to exhibit higher rates of joint surgery, likely due to delayed treatment initiation, cumulative joint damage, and age-related factors such as weaker joints (decreased joint resilience). Surgical intervention is typically reserved for severe joint pain or functional impairment that cannot be managed via non-surgical approaches during the “end stage”, with procedures tailored to each patient’s specific condition to relieve pain and restore joint function [17,263].

One of the most common surgical interventions in RA patients is tenosynovectomy, primarily performed on the hands to alleviate pain, prevent further damage, and restore tendon function [263,264]. Radiosynovectomy, a minimally invasive alternative approach often applied to the knee, helps minimize inflammation and prevent joint deterioration, particularly in cases where multiple joints are affected. It involves injecting a small dose of radioactive material, known as radiopharmaceuticals, into the affected joint(s), thereby targeting the synovial lining. Rather than undergoing multiple separate procedures, this method enables explicitly targeted treatment in various joints within the same session, thereby improving symptom relief and overall functional mobility [263,265]. Similarly, traditional synovectomy, commonly performed on the shoulder’s rotator cuff, knee, or hip, enhances joint mobility and tendon recovery [263,266,267].

Another surgical option is osteotomy, which involves the surgical realignment of weight-bearing bones, mainly in cases of knee deformities (e.g., valgus or varus), so as to reduce pain and improve mobility [263,268,269]. Furthermore, soft-tissue release procedures help address contractures and restore movement, although these approaches have become less common due to the emergence of more effective non-surgical treatments for RA [263,270]. A more recent surgical technique, joint fusion (arthrodesis), is performed on the ankle, wrist, thumb, or cervical spine, offering stability and pain relief in cases of severe joint degeneration [263,267]. Additionally, small-joint implant arthroplasty is commonly used to improve hand function and also reduce pain [263,271,272]. Total joint replacement (arthroplasty), applied to the shoulder, elbow, wrist, hip, knee, or ankle, remains a highly effective approach for managing pain and restoring joint function [263,273,274]. Metatarsal-head excision arthroplasty, a widely used procedure in RA management, is particularly effective in relieving severe forefoot pain induced by systemic inflammation [263,267,268,275]. Given the broad range of surgical options available for RA management, the choice of procedure depends on multiple factors, such as the affected joint, disease severity, and the patient’s overall health status [17,263]. Table 1 summarizes the mechanisms of RA-associated thrombosis and the related therapeutic approaches discussed in Section 3.6, Section 3.6.1, Section 3.6.2, Section 4, Section 4.1, Section 4.2, Section 4.3, Section 4.3.1, Section 4.3.2, Section 4.3.3, Section 4.4, Section 4.4.1, Section 4.4.2, Section 4.4.3, Section 4.4.4, Section 4.5 and Section 4.6.

## 5. Limitations, Future Perspectives, and Potential Treatment Solutions

Despite the significant advancements in RA-targeted therapies, several gaps, limitations, and challenges persist, highlighting the urgent need for further improvement. Key issues include the lack of personalized treatment approaches, the scarcity of pediatric-focused studies for rheumatologic conditions [286], and the increased risk of infections and complications, especially with the long-term use of bDMARDs, GCs, and JAK inhibitors [17]. Additional concerns involve treatment fluctuations (e.g., fatigue, pain, loss of function) [17,38,140], the high cost of bDMARDs and JAK inhibitors [287], incomplete understanding of RA’s pathophysiology, and treatment unresponsiveness over time [35,139]. Moreover, limited data on the role of the microbiome and genetics [147], along with an inadequate assessment of mental health and daily function in RA patients, suggest substantial room for progress [17,38,140].

Preventative strategies are now recognized as crucial in RA management, with four distinct levels of prevention [17]: primary prevention, which focuses mainly on restricting RA onset by mitigating risk factors; secondary prevention, which focuses on early identification and intervention to slow disease progression; tertiary prevention, which is capable of minimizing long-term damage and disability; and finally, clinical prevention, which reduces complications and relapses [17,288]. RA management has extensively developed over the past few years, where the focus has shifted towards the improvement of therapeutic interventions and bettering early diagnosis for RA prevention [17,288].

Although central RA-associated inflammatory mechanisms have been elucidated, the underlying immunological and thrombo-inflammatory mechanisms remain incompletely understood. Consequently, experimental models and novel therapeutic agents are continuously being evaluated in clinical trials. As Radu and Bungau [17] and Huang et al. [195] have proposed, promising investigational therapies include JAK inhibitors (e.g., filgotinib, peficitinib) [289,290], p38 MAPK inhibitors (e.g., RO4402257 (pamapimod)), α–chemokine receptor ½ (CXCR1/2) inhibitors (e.g., repertaxin) [291], MMP-9 inhibitors (e.g., andecaliximab) [292], granulocyte-macrophage colony-stimulating factor (GM-CSF) inhibitors (e.g., otilimab) [293], G-protein-coupled receptor kinase 2 (GRK2) inhibitors (e.g., paroxetine) [294,295], antagonists of CD20 on T cells (e.g., ofatumumab) [296], IL-10 inhibitors (dekavil) [297], and IL-15, -17, and -18 inhibitors in many different clinical phases. Given the interplay between RA and thromboembolism, conventional treatments, including GCs [289], JAK [201], and IL-17/23 inhibitors [276,298,299], may increase VTE risk by promoting inflammation, hypercoagulation, and endothelial dysfunction. Therefore, safer alternatives, including tsDMARDs and bDMARDs, carefully monitored regimens, as well as personalized treatment plans, particularly for patients with high CVD risk, are essential for targeting RA-associated thrombosis [9,17,282].

Small-molecular metabolite targets like prostaglandins, T_X_A_2_, PAF, leukotriene B4 receptor, cannabinoid receptors, epigenetic regulators (i.e., DNA, RNA, or histone alterations), and protein agents (i.e., p38 MAPK, GRK2, or GM-CFS agents) have shown promising potential in RA treatment, especially when combined with tsDMARDs or bDMARDs [195]. Regarding inflammatory arthritis, significant attention has been drawn to TLRs, mainly TLR4 and TLR5, as key players in RA pathogenesis. Emerging anti-TLRs-based therapies, such as those targeting tenascin C, have displayed notable antirheumatic potential [300]. Additionally, mesenchymal stem cells (MSCs) have been shown to interact with RA fibroblast-like synoviocytes (RA-FLSs), thereby suppressing pro-inflammatory markers and reducing thrombotic activity [301]. Another promising avenue is the use of DCs—particularly DCs generated ex vivo—which have demonstrated autoimmune-suppressive capabilities in preliminary studies, although further research is needed [302]. Beyond pharmacological interventions, personalized rehabilitation plans play a pivotal role in managing chronic RA symptoms and improving patients’ quality of life [286,303]. Moreover, artificial intelligence (AI) has emerged as a powerful tool for early diagnosis, RA prediction, and prevention, as well as the optimization of personalized treatment [286,303]. Finally, to address poor solubility, permeability, and bulky molecular structures of many RA therapies—particularly oral treatments—the integration of nanotechnology into drug delivery systems has gained widespread support. Nanoformulations have the potential to enhance drug bioavailability and reduce side effects, making them a promising treatment for RA [142,304,305].

A reduction in RA-identified indices and immunological markers has been observed with triterpene treatment, as triterpenes exhibit antioxidant, anti-inflammatory, and anti-thrombotic properties [306]. The main objective of non-pharmacological RA approaches is to alleviate anxiety, depression, and pain while enhancing mobility, mental health, and daily functioning among patients [306]. PUFAs, particularly ω-3 fatty acids like DHA and EPA, have garnered attention for their potential to mitigate neurological disorders, including anxiety and depression. A recent meta-analysis evaluating PUFA efficacy in treating depression highlighted that a 2:1 EPA: DHA ratio provided the most potent antidepressant effects [38]. Moreover, oral supplementation with ω-3 PUFAs has been associated with a lower incidence of RA and reduced RA severity [283,307]. Of particular interest is the exploration of polar amphiphilic PUFA forms, where PUFAs are bound to polar lipids (esterified either in phospholipids or glycolipids). This modification has shown promise in enhancing bioavailability and increasing activity at lower doses by improving interactions with thrombo-inflammatory mediators and reducing half-maximum inhibitory concentration (IC_50_) values [308]. Investigating these polar-lipid-bound PUFAs could offer therapeutic benefits and better inflammatory control in RA patients [283,309,310].

It is essential to clarify that although ω-3 PUFAs have shown promising potential in RA treatment, current formulations—such as fatty esters in triglycerides or monoesters—are predominantly neutral forms with low bioavailability due to their hydrophobic nature. As a result, high doses are required to achieve therapeutic effects, which not only reduce efficacy but also increase the risk of adverse effects and complicate long-term adherence to supplementation. A more promising approach involves the utilization of ω-3 PUFAs bound to polar lipids (e.g., glycolipids or phospholipids), which have demonstrated higher bioavailability and bioactivity and reduced side effects [283,309,310]. In the context of RA treatment, these polar forms deliver enhanced therapeutic benefits at significantly lower doses. Consequently, future studies should investigate the anti-inflammatory efficacy and safety profile of polar-lipid-bound ω-3 PUFAs compared to traditional forms. The shift toward polar lipids marks a notable advancement in ω-3 supplementation, revolutionizing the management of chronic inflammatory diseases (e.g., RA) [308,311].

Targeted RA therapies have significantly advanced disease management, particularly in reducing systemic inflammation, a key driver of RA-related thrombosis. By modulating inflammatory pathways and markers, such as cytokines, coagulation factors, and fibrinolytic agents, these therapies not only alleviate RA symptoms but also help mitigate the risk of thrombosis. Notably, enhancing the efficacy of significant class inhibitors, such as TNF-α, IL-6, and the JAK-STAT pathway, while minimizing their reported side effects could lead to more effective prevention and a more significant reduction in both the inflammatory and thrombotic burdens in RA patients. However, further studies are necessary to evaluate the long-term effects of these treatments on the risk of RA-induced thrombosis and to identify patient-specific factors that may influence therapeutic outcomes. A promising avenue lies in combining targeted therapies with antithrombotic strategies as part of a personalized treatment approach. Such tailored interventions dedicated to RA management have the potential to strike a balance between efficacy and safety, ultimately improving patient outcomes and reducing disease-related complications associated with RA [19,38,165].

## 6. Conclusions

In this comprehensive review, arterial and venous thrombosis were thoroughly examined, along with their relationship to RA, which is considered an inflammatory condition. Various alternatives to traditional therapeutics, including low-MW heparin, anticoagulants, csDMARDs, GCs, and NSAIDs, are used to manage thrombosis in RA. However, they come with risks like bleeding and adverse effects. Emerging therapies such as bDMARDs, tsDMARDs, MSCs, ω-3 PUFAs, and other novel agents show promise in improving treatment efficacy while reducing symptoms like pain and inflammation. Future research should focus on the intersection of AI and nanotechnology to develop safer, more accessible, personalized, and targeted RA–thrombosis treatments with fewer side effects.

## Figures and Tables

**Table 1:** Mechanisms of RA-associated thrombosis and related therapeutic approaches.

Mechanisms of RA-Associated Thrombosis ^1^	Description	Therapeutic Approaches	Adverse Effects and Risks	References
Endothelial Injury	• Endothelial dysfunction occurs early in RA, increasing cell activation, permeability, leukocyte–platelet adhesion, and monocyte–endothelium interactions • It has been linked to increased inflammation and atherosclerosis • Elevated VWF and PAI-1 reinforce the RA–thrombosis link and contribute to arterial and venous thrombosis	• TNF-α (i.e., etanercept, adalimumab) or IL inhibitors reduce endothelial activation • GC tapering as a “bridge therapy” in early RA treatment before DMARD use • Antiplatelet therapies like aspirin prevent platelet adhesion induced by endothelial dysfunction • Methotrexate may also improve endothelial function in early RA patients	• Risk of infections, tuberculosis, lymphoma, thromboembolism, and VTE during anti-TNF-α drug use • GC-induced endothelial injury resulting from decreased NO production and vascular relaxation	[8,38,80,94,160,162,163,164,173,229,276,277]
Hypercoagulability	• Chronic inflammation elevates procoagulant factors (e.g., fibrinogen, thrombin), reduces anticoagulants, and suppresses fibrinolysis • Pro-inflammatory markers like IL-6 and TNF-α are linked to RA-related prothrombotic activity • Intravascular activation leads to a hypercoagulable state, subsequently increasing VTE risk	• GCs, TNF-α, IL-6 (i.e., tocilizumab), and JAK inhibitors reduce the risk of thromboembolism by controlling systemic inflammation and RA-related hypercoagulability • Anticoagulants like warfarin and DOACs (e.g., dabigatran, rivaroxaban) prevent clot formation by inhibiting thrombin and factor Xa	• GCs have also been connected to increased levels of procoagulant factors and VTE risk • Increased bleeding risk, especially with prolonged use of anticoagulants • Risk of tuberculosis, thromboembolism, and VTE during anti-TNF-α drug use	[38,61,165,166,167,173,256,278,279]
Venous Stasis	• Plasma hyperviscosity due to elevated CRP, FVII, fibrinogen, and VWF, combined with prolonged immobility during RA flare-ups, contributes to impaired venous circulation and thrombosis	• Thromboprophylaxis with anticoagulants (e.g., low-MW heparin) in high-risk patients alongside physiotherapy to improve circulation • Anti-TNF-α medication (i.e., etanercept) and JAK inhibitors (e.g., B cell depleters like rituximab) reduce the inflammatory response by suppressing plasma hyperviscosity	• Risk of bleeding with anticoagulants; immobility may increase clot formation despite treatment • Risk of tuberculosis, thromboembolism, and VTE during anti-TNF-α drug use	[8,35,38,86,168,169,170,172,173,176,177]
Medication-induced Risk	• JAK inhibitors (e.g., baricitinib, tofacitinib) have been correlated with thromboembolic events in RA patients, potentially increasing VTE risk • Similarly, GCs, TNF-α and IL inhibitors have been linked to several thromboembolic events and VTE onset	• Risk assessment, personalized treatment, and VTE monitoring are essential for RA patients receiving JAK inhibitors, GCs, or bDMARDs	• Increased risk of thrombosis, infections, lymphoma, and potential CV events	[38,173,177,178,179,227,245,261]
Platelet Activation	• ADP, T_X_A_2_, and thrombin amplify platelet activation, enhancing aggregation and clot formation • T_X_A_2_ plays a critical role in RA by sustaining inflammation through an auto-regulatory loop • Such factors aggravate thrombo-inflammation and impair fibrinolysis	• Combinations of anti-thrombotic medication, antiplatelets, anticoagulants (i.e., warfarin, low-MW heparin, DOACs), and/or fibrinolytic drugs may be beneficial • Lipid-metabolite-based drugs are appealing solutions • NSAIDs (e.g., COX-1/2 inhibitors like celecoxib and rofecoxib) and GCs reduce inflammation, arterial clot formation, and platelet aggregation • P2Y12 inhibitors (e.g., clopidogrel) and T_X_A_2_ blockers prevent platelet activation and reduce thrombotic manifestations • csDMARDs (i.e., methotrexate, hydroxychloroquine, leflunomide) reduce fibrinogen and coagulation inhibitors • bDMARDs (i.e., IL-6 or TNF-α inhibitors) and tsDMARDs (i.e., JAK inhibitors) have offered better platelet aggregation reduction than csDMARDs	• NSAIDs are associated with an increased risk of GI irritation, renal impairment, bleeding, pain, and CVDs • GCs have been linked to weight gain, water retention, muscle weakness, and insulin resistance • Increased risk of bleeding and impaired clotting function with antiplatelet drugs • High risk of CVD events like VTE onset, hepatotoxicity, and retinal toxicity following csDMARDs administration • Risk of tuberculosis, thromboembolism, and VTE during anti-bDMARDs and tsDMARDs use	[8,38,62,91,99,167,173,203,205,205,206,233,254,278]
PAF Activation	• Elevated PAF levels exacerbate inflammation and contribute to endothelial dysfunction, platelet hyperactivity, and increased oxidative stress, driving RA progression and thrombus formation • RA patients exhibit elevated circulating platelet and PAF activity and are associated with leukocyte activation and systemic inflammation aggravation	• Anti-PAF and anti-PAF-R drugs, alongside nature-derived polar lipids, exhibit anti-thrombotic and anti-inflammatory properties	• Limited clinical data on anti-PAF therapies; potential off-target effects	[27,62,109,173,185,195,196,197,199]
Inflammatory Cytokines	• TNF-α, IL-1β, and IL-6 are pro-inflammatory cytokines that drive systemic inflammation, reduce natural anticoagulation mechanisms, and contribute to endothelial injury and thrombotic events	• csDMARDs exert high anti-inflammatory effects but are currently pointed out as less effective than bDMARDs and tsDMARDs • bDMARDs (e.g., TNF-α inhibitors, IL-6 blockers) and tsDMARDs (e.g., JAK inhibitors) target inflammatory pathways to mitigate thrombosis risk observed in RA patients	• Risk of tuberculosis, lymphoma, thromboembolism, and VTE during bDMARDs and tsDMARDs use • Increased susceptibility to infections and immune suppression	[38,143,144,158,159,160,177,201,232,233,238,244,245,246,249,250,251,256,259,261,262,280,281,282]
Oxidative Stress	• Increased oxidative stress leads to endothelial damage, promoting platelet activation and enhancing thrombogenesis in RA	• Antioxidants and anti-inflammatory agents (e.g., ω-3 PUFAs) reduce oxidative stress and improve vascular health	• Potential GI discomfort and interactions with other medications	[38,105,107,108,147,157,174,175,176,283]
Surgery-related Risks	• Joint damage and immobility due to severe RA increases the risk of venous stasis and thrombotic events, particularly post-surgery • Surgical interventions (e.g., radiosynovectomy, traditional synovectomy, osteotomy, soft-tissue release, arthroplasty) aim to relieve pain, restore function, and prevent further joint damage in advanced RA stages	• Surgery combined with medical therapy, including bDMARDs or tsDMARDs, aims to enhance post-surgical outcomes and minimize thrombosis risk	• Increased risk of thrombosis due to surgery-induced vascular injury, prolonged immobility, and post-operative inflammation • Age-related factors and cumulative joint damage may further elevate the risk	[17,133,208,263,266,271,274,275,284,285]

^1^ Summary of the mechanisms and therapeutic approaches included in Section 3.6, Section 3.6.1, Section 3.6.2, Section 4, Section 4.1, Section 4.2, Section 4.3, Section 4.3.1, Section 4.3.2, Section 4.3.3, Section 4.4, Section 4.4.1, Section 4.4.2, Section 4.4.3, Section 4.4.4, Section 4.5 and Section 4.6.
